# The Ketogenic Diet in Type 2 Diabetes and Obesity: A Narrative Review of Clinical Evidence

**DOI:** 10.3390/nu18030397

**Published:** 2026-01-25

**Authors:** Julia Kilian, Dominika Szlęzak, Malgorzata Tyszka-Czochara, Elżbieta Filipowicz-Popielarska, Patrycja Bronowicka-Adamska

**Affiliations:** 1 Chair of Medical Biochemistry, Faculty of Medicine, Jagiellonian University Medical College, 7c Kopernika St., 31-034 Cracow, Poland; julia.kilian@student.uj.edu.pl (J.K.); dominika.szlezak@uj.edu.pl (D.S.); 2 Faculty of Pharmacy, Jagiellonian University Medical College, 9 Medyczna St., 30-688 Cracow, Poland; malgorzata.tyszka-czochara@uj.edu.pl; 3 Department of Medicine, Faculty of Medicine and Health Sciences, University of Applied Sciences, 8 Mickiewicza St., 33-100 Tarnów, Poland; empopielarska@gmail.com

**Keywords:** diet, obesity, type 2 diabetes

## Abstract

Type 2 diabetes mellitus (T2DM) and obesity represent a growing global public health challenge, strongly associated with excess body weight, unhealthy dietary habits, and a sedentary lifestyle. The ketogenic diet (KD), characterized by very low carbohydrate intake, moderate protein intake, and high fat consumption, induces a metabolic state known as ketosis, in which the body switches from glucose to fat as its primary energy source. KD has gained increasing interest as a strategy to improve glycemic control, reduce body weight, and improve lipid profiles in individuals with obesity and T2DM. The purpose of this narrative review is to summarize the current scientific evidence on the effects of KD on key metabolic parameters, including blood glucose levels, glycated hemoglobin (HbA1c), body weight, and body composition. The analysis is based on peer-reviewed articles retrieved from PubMed, Embase, and Scopus with particular emphasis on clinical studies that provide robust evidence on the efficacy and safety of KD in the treatment of metabolic disorders.

## 1. Introduction

The increasing prevalence of diabetes and obesity constitutes a major public health concern in the 21st century, both at the individual and population levels [1,2]. The diseases are regarded as the main constituents of the metabolic syndrome, a cluster of interrelated metabolic disorders, including abdominal obesity, insulin resistance, dyslipidemia, and hypertension, which collectively increase the risk of type 2 diabetes and cardiovascular disease. The etiology of these conditions is multifactorial, involving genetic predisposition, environmental influences, and lifestyle-related factors, particularly inadequate diet patterns [3]. Conventional therapeutic interventions often have limited efficacy, and the incidence of metabolic disorders continues to rise. Therefore, there is growing interest in novel dietary strategies that can help manage disease and prevent disease progression [4,5]. The ketogenic diet (KD), defined as a nutritional approach characterized by very low carbohydrate intake and high fat consumption, has garnered increasing scientific attention due to its potential to improve various metabolic parameters [6,7]. By inducing a state of ketosis, this dietary model shifts energy metabolism in a manner that can result in reduced glycemia, increased insulin sensitivity, weight loss, and improved lipid profiles [8,9]. Nevertheless, the long-term safety, sustainability, and efficacy of this dietary approach remain the subject of ongoing investigation [10,11]. The purpose of the present study is to provide a comprehensive and critical review of current scientific evidence on the application of the ketogenic diet in the management of type 2 diabetes and obesity, including both primary interventional studies and already published reviews and meta-analyses, in order to offer a detailed overview of current evidence on the effects of the ketogenic diet on type 2 diabetes and associated obesity. Particular emphasis is placed on elucidating the underlying mechanisms of action, evaluating clinical outcomes reported in recent studies, and assessing potential health risks associated with long-term adherence to this diet [11]. In addition, this article seeks to determine whether the ketogenic diet may constitute a viable and evidence-based component of comprehensive metabolic disease management strategies in contemporary clinical practice [12].

## 2. Materials and Methods

This article was prepared based on a critical analysis of the literature sourced from databases such as PubMed, Embase, and Scopus. Articles were searched and analyzed using the following subject keywords: diet, ketogenic diet, ketosis, low-carbohydrate diet, nutrition, definition, obesity, diabetes, diabetes mellitus, type 1 diabetes, type 2 diabetes, gestational diabetes, guidelines, carbohydrate intake, mechanism, outcome, benefits, side effects. In addition, a block search strategy was used to systematically identify relevant studies, using the logical operators “AND” and “OR”. After searching the databases, duplicate records were removed so that each article was represented only once. A rigorous selection procedure was then performed using inclusion and exclusion criteria: Inclusion criteria: research on the effect of the ketogenic diet on obesity and/or type 2 diabetes, clinical trials, systematic reviews, meta-analyses, and experimental studies in humans or animal models relevant to metabolism. Exclusion criteria: articles on other types of diets or nutritional interventions not related to the ketogenic diet (reason 1), publications with unclear or insufficiently described methodology (reason 2), and articles not reviewed by peer reviewers (e.g., conference abstracts, comments, letters to the editor) (reason 3).

**Scheme 1:**
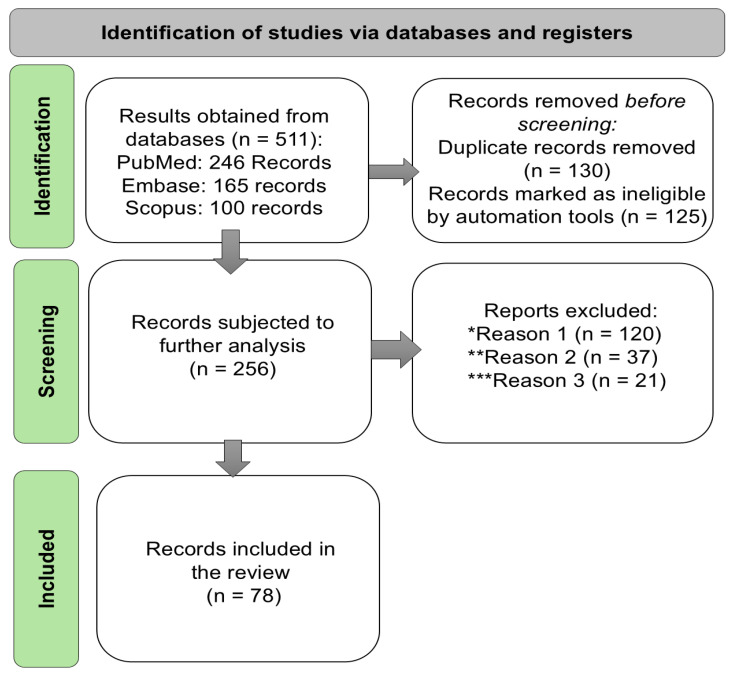
Schematic representation of the literature selection process for this review, showing the screening and eligibility assessment of the retrieved articles. *Reason 1: articles on other types of diets or nutritional interventions not related to the ketogenic diet; **Reason 2: publications with unclear or insufficiently described methodology; ***Reason 3: articles reviewed by non-peers (e.g., conference abstracts, comments, letters to the editor). Adapted from PRISMA 2020 (https://www.prisma-statement.org/prisma-2020-flow-diagram; accessed on 18 November 2025).

Eventually, selected studies on the impact of the KD as a metabolic therapy for obesity and type 2 diabetes were included in the analysis. The literature selection process is presented in the PRISMA scheme (Scheme 1), which visually illustrates the step-by-step filtering of records, increasing the transparency and reproducibility of the review.

## 3. Nutritional and Metabolic Mechanisms of the KD in T2DM and Related Obesity-Driven Metabolic Disorders

### 3.1. Mechanistic Basis of the Ketogenic Diet and Its Implementation in Clinical Practice

**Figure 1:**
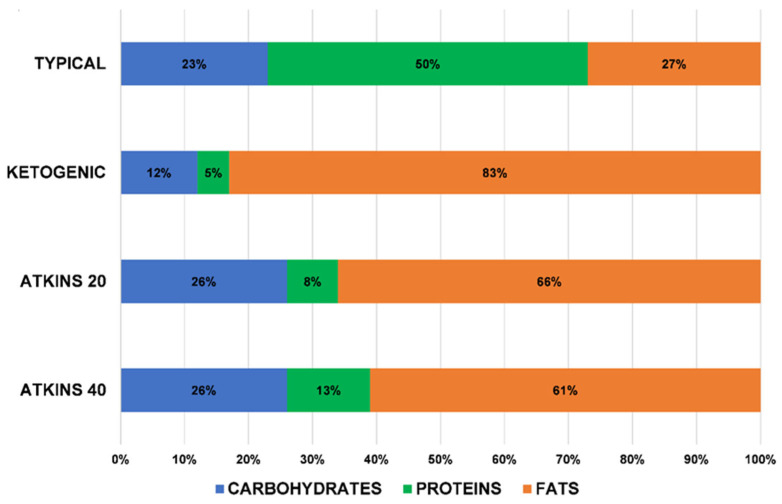
The approximate differences in macronutrient distribution across four dietary patterns: a typical Western diet, the ketogenic diet, the Atkins 20 diet, and the Atkins 40 diet. The author’s own elaboration is based on data from the literature [13].

**Figure 2:**
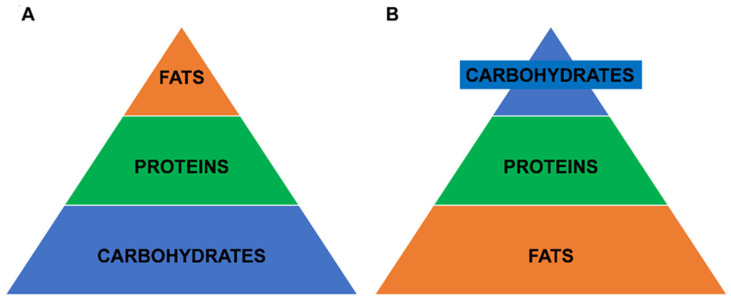
Comparison of macronutrient distribution in the traditional food pyramid (**A**) and the ketogenic diet (**B**). The author’s own elaboration is based on data from the literature [17].

The ketogenic diet represents a specific form of a low-carbohydrate diet in which carbohydrate intake is restricted to a level that induces and maintains a state of nutritional ketosis. This diet is characterized by a high proportion of fat in the energy supply, moderate protein intake, and a very low carbohydrate intake. In a wider dietary context, KD is classified as a low-carbohydrate diet, which includes various nutritional models, such as the low-carb, high-fat diet or the Atkins diet, some of which, depending on the degree of carbohydrate reduction, can lead to a ketosis state [13,14]. It should be emphasized that low-glycemic index diets are not inherently ketogenic. Although such dietary approaches can attenuate postprandial glycemic excursions, they generally do not induce or sustain nutritional ketosis due to insufficient carbohydrate restriction. KD includes various variants with different macronutrient ratios, all of which aim to induce ketosis by significantly reducing carbohydrates and increasing fat intake [15]. Figure 1 shows a comparison of the proportions of carbohydrates, proteins, and fats in four dietary models: a typical Western diet, the ketogenic diet, the Atkins 20 diet, and the Atkins 40 diet. The infographics illustrate a clear decrease in carbohydrate intake and an increase in fat intake in low-carbohydrate diets, especially the ketogenic diet. The KD model, most commonly used in scientific studies, restricts carbohydrate intake to less than 30–50 g per day. This causes the body to enter a state of ketosis in a few days [13,16]. In contrast to the conventional dietary pyramid recommended by nutritional guidelines, the ketogenic diet prioritizes fat as the main energy source while minimizing carbohydrates (Figure 2) [17].

Four main types of KD can be identified: the classic ketogenic diet, based on long-chain triglycerides used clinically, is characterized by a 4:1 (g/g) ratio of fat to protein and carbohydrates. It is effective but difficult to prepare and is often poorly accepted in terms of taste [18]. The MCT diet, developed in 1971, uses MCT oil as the main source of fat, allowing greater flexibility in the selection of macronutrients and stronger ketogenesis, although it might cause gastrointestinal discomfort [18]. The modified Atkins diet (MAD) is actually a milder and less strict version of KD—it does not require strict food weighing or calorie restriction. The diet initially limits carbohydrates to 10–15 g/day, with the possibility of increasing this to 20 g/day [18]. In turn, a low-glycemic index diet involves moderate carbohydrate restriction with the elimination of foods with a GI > 50, focusing on glycemic stabilization as a potential mechanism of neuroprotective action [18]. In recent decades, ketogenic diets, especially the Atkins diet, have gained considerable popularity as nutritional strategies that lead to secondary ketosis [15]. The conventional KD are based on a ratio of fat to total carbohydrates and protein of 4:1 or 3:1. The 4:1 ratio provides 90% of energy from fat, 2–4% of energy from carbohydrates, and 6–8% of energy from protein, while the 3:1 ratio provides 85–90% of energy from fat, 2–5% of energy from carbohydrates, and 8–12% of energy from protein [16]. Compared with nutritional therapy guidelines, healthy eating principles recommend limiting excessive carbohydrate intake while maintaining fat intake at 20–35% of total energy, with emphasis on reducing saturated fats. In contrast, the ketogenic diet is characterized by a high intake of fats from foods such as meat, fish, eggs, dairy products, nuts, seeds, avocados, and non-starchy vegetables. The diet restricts major carbohydrate sources, including grain products (e.g., pasta and bread), starchy vegetables, and most fruits. Alcohol is generally discouraged, although beverages with negligible carbohydrate content may be consumed in limited amounts.

**Scheme 2:**
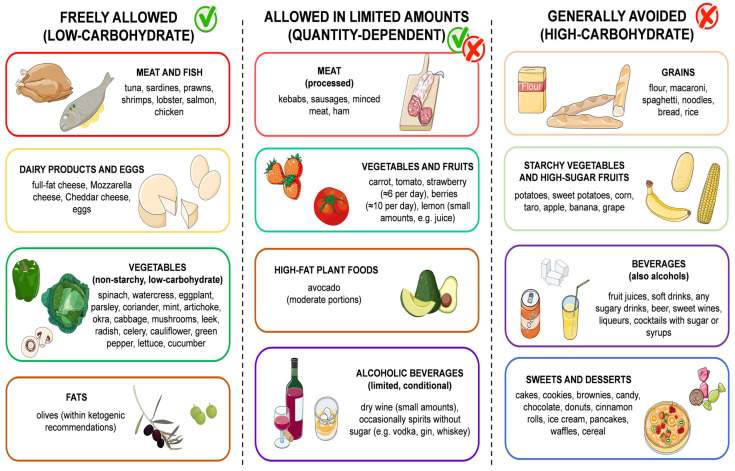
Food categories within a ketogenic diet pattern, depending on carbohydrate content and portion size [19]. The scheme is an original work of the author and was created using Servier Medical Art graphics (https://smart.servier.com/, accessed on 2 November 2025).

Reduced carbohydrate intake can result in lower dietary fiber consumption, as many fiber-rich foods are restricted. However, adequate fiber intake can be partially maintained through the careful selection of low-carbohydrate vegetables. Some fruits, particularly berries, may be included in small quantities; therefore, the inclusion of food within KD largely depends on carbohydrate content and portion size (Scheme 2).

### 3.2. Biochemical and Metabolic Basis of the Ketogenic Diet

**Scheme 3:**
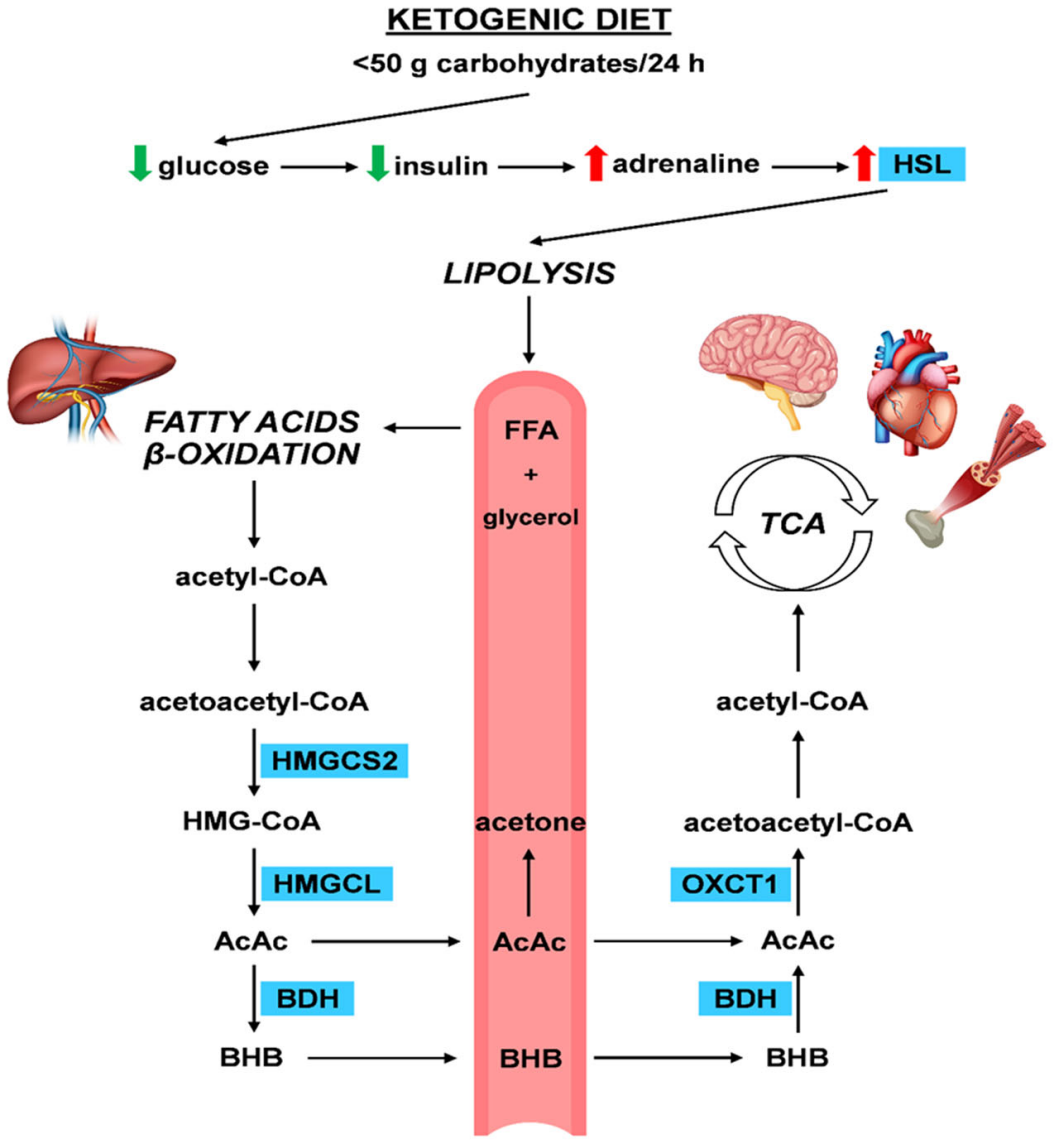
Biochemical basis of ketosis: regulation of lipolysis, β-oxidation, and ketone body production during a ketogenic diet. Abbreviations: AcAc—Acetoacetate, Acetyl-CoA—Acetyl coenzyme A, BHB—β-Hydroxybutyrate, BDH—β-Hydroxybutyrate dehydrogenase, FFA—Free fatty acids, HMG-CoA—3-Hydroxy-3-methylglutaryl-CoA, HMGCL—3-Hydroxy-3-methylglutaryl-CoA lyase, HMGCS2—3-Hydroxy-3-methylglutaryl-CoA synthase 2, HSL—Hormone-sensitive lipase, OXCT1—Succinyl-CoA:3-ketoacid CoA transferase 1, and TCA—Tricarboxylic acid cycle. The author’s own elaboration based on data from the literature, created using Servier Medical Art graphics (https://smart.servier.com/, accessed on 2 November 2025).

Implementing KD requires a marked reduction in carbohydrate intake, typically below 50 g/day, and during the initial adaptation phase, even to approximately 20 g/day [20]. Reduced glucose availability lowers insulin levels and increases adrenaline secretion, thus activating hormone-sensitive lipase and promoting lipolysis. This process leads to the release of free fatty acids, which undergo β-oxidation in hepatocyte mitochondria. Increased utilization of oxaloacetate for gluconeogenesis limits its availability for the tricarboxylic acid cycle, resulting in accumulation of acetyl-CoA and subsequent production of ketone bodies—acetoacetate (AcAc), β-hydroxybutyrate (BHB), and acetone [21]. Ketogenesis occurs predominantly in hepatocytes and is regulated by 3-hydroxy-3-methylglutaryl-CoA synthase 2 (HMGCS2). Ketone bodies are released into the circulation and utilized by extrahepatic tissues as alternative energy substrates, where they are converted back to acetyl-CoA and enter the tricarboxylic acid cycle. The liver does not utilize ketone bodies due to the absence of 3-oxoacid CoA transferase (OXCT1) [22]. Physiological ketosis induced by a ketogenic diet represents an adaptive metabolic state and should be distinguished from pathological ketoacidosis observed in type 1 diabetes [22]. The regulation of lipolysis, β-oxidation, and ketone body production during a ketogenic diet is summarized in Scheme 3.

### 3.3. The Ketogenic Diet as a Metabolic Intervention in Diabetes Management

T2DM is the most prevalent form of diabetes and constitutes a major contributor to global morbidity and mortality, primarily due to its numerous microvascular and macrovascular complications. A key non-pharmacological strategy capable of reducing glycemic variability and improving metabolic control is lifestyle modification, with particular emphasis on dietary interventions. The American Diabetes Association (ADA) report recommends a low-carbohydrate diet as an appropriate dietary intervention for patients with diabetes or prediabetes. One variant of low-carbohydrate diets is the ketogenic diet. The KD can be risky for patients with type 1 diabetes (T1DM), especially when using SGLT-2 inhibitors, and requires medical supervision [23]. The use of this diet improves metabolic status in hyperglycemic patients by lowering blood glucose levels and increasing tissue sensitivity to insulin [19]. The beneficial effect of KD in patients with diabetes is due to its characteristics. Minimizing carbohydrate intake is associated with reduced absorption of simple sugars, leading to lower blood glucose levels and lower glycemic fluctuations [23]. Due to the well-established comorbidity between T2DM and obesity, the metabolic and clinical effects of the KD on T2DM cannot be considered in isolation; rather, they are intrinsically related to its mechanisms of action and therapeutic impact on obesity. However, the molecular mechanisms connecting obesity with the development of type 2 diabetes remain partially unknown. There is evidence that ectopic lipid deposition in non-adipose tissues, including the liver, skeletal muscles, and pancreas, disrupts normal insulin signaling, promoting insulin resistance, hyperinsulinemia, and progression of metabolic syndrome [24,25]. Research by Lean et al. [26] has confirmed that weight loss alone can result in remission of diabetes in nearly 46% of patients after 12 months. However, this does not solve the problem of diabetic patients who are not overweight [17]. KD is well-suited for achieving rapid and safe weight loss, which contributes to, among other things, improvements in blood glucose control [27]. The meta-analysis [28] examining the effects of the KD on weight loss, glycemic control, and lipid profiles in overweight individuals with type 2 diabetes demonstrated that KD was a more effective intervention than other dietary approaches. This dietary intervention was also associated with a reduction in waist circumference—a marker of central obesity and an important risk factor for the progression of diabetes and its complications. Severe restriction of carbohydrate intake leads to reduced monosaccharide absorption, lower blood glucose levels, and diminished glycemic fluctuations, indicating a beneficial effect on glucose metabolism in patients with T2DM [29,30]. Findings from the same meta-analysis [28] demonstrated that the ketogenic diet induces a greater reduction in HbA1c levels compared with other dietary interventions. All included studies lasted at least three months; therefore, the differences between baseline and final HbA1c values reliably reflect the effectiveness of the dietary intervention. Although this analysis did not show significant changes in fasting insulin levels, a tendency toward their reduction was observed in the KD group. The results of the meta-analysis also suggest that the KD may contribute to improvements in lipid profile, including decreased triglyceride levels and increased HDL concentrations (Table 1). Reducing carbohydrate intake decreases fluctuations in blood glucose levels. This is important, as many individuals with diabetes who are not overweight also require effective glycemic control.

### 3.4. The Effect of the Ketogenic Diet on Glycemic Control in Patients

Nutritional therapy plays a key role in the optimal management and prevention of diabetes. The ketogenic diet is one of the dietary approaches used to modify diabetes-related risk factors, including obesity and hyperglycemia [4,15,30]. Narrative review of randomized controlled trials conducted by Alluwyam and Estrella [31] demonstrated that KD is associated with significant improvements in glycemic control, including reductions in HbA1c, as well as favorable effects on body weight and insulin sensitivity. The authors also highlighted the potential role of ketogenic diets in reducing the risk of T2DM-related complications (Table 1). In contrast, Choy et al. [19] in a meta-analysis of randomized controlled trials, reported no significant differences between ketogenic and control diets with respect to improvements in glycemic control or body weight in patients with T2DM. A systematic review and meta-analysis by Yuan et al. [32] demonstrated that KD significantly improves glycemic control and insulin resistance in patients with type T2DM. The intervention was also associated with beneficial changes in lipid metabolism, supporting the role of carbohydrate restriction as an effective nutritional strategy in the management of T2DM (Table 1). Research on KD has mainly focused on T2DM, as studies on T1DM are limited due to the increased risk of hypoglycemia and diabetic ketoacidosis [22]. Some case reports suggest potential benefits of KD in T1DM under careful medical supervision, but long-term adherence can be challenging, particularly for children and adolescents. KD requires precise meal planning, strict macronutrient monitoring, and continuous medical supervision. Long-term adherence is difficult, with only 50% of adult T1DM patients maintaining the diet after four years [33]. Three randomized clinical trials and an online pilot study demonstrated that low- or very-low-carbohydrate ketogenic diets (LCKD or VLCKD) improve glycemic control, reduce body weight, and lower triglyceride levels in individuals with type 2 diabetes. Westman et al. [34] conducted a 24-week RCT with 84 participants, comparing an LCKD (<20 g carbohydrates/day) with a low-glycemic, reduced-calorie diet (LGID); the LCKD resulted in greater reductions in HbA1c, weight loss, increased HDL, and a reduction or discontinuation of antidiabetic medications. Goday et al. [35], in a 4-month RCT with 89 participants, showed that a VLCKD (<50 g carbohydrates/day) combined with lifestyle support led to greater reductions in body weight and waist circumference and better glycemic control compared with a standard low-calorie diet, with mild adverse effects. Saslow et al. [36] conducted a 32-week online pilot study with 25 participants, in which VLCKD plus lifestyle modifications improved HbA1c, body weight, and triglyceride levels, increased the proportion of participants achieving therapeutic targets, and resulted in lower dropout rates compared with the ADA “Create Your Plate” online program (Table 2).

### 3.5. Ketogenic Diet as an Effective Approach to Treating Obesity and Reducing Body Weight

A major motivation for following a ketogenic diet is its proven effectiveness in promoting weight loss. Accomplishing weight loss requires an effective metabolic change toward nutritional ketosis. The initial weight loss is mainly due to water loss, which occurs as a result of glycogen depletion. This water was then restored with the reintroduction of carbohydrates and the replenishment of glycogen [13]. In a long-term study involving 64 obese individuals (BMI > 30) [4], participants followed a dietary protocol consisting of less than 20 g of carbohydrates per day for the first 12 weeks, which then gradually increased to 40 g per day. Body weight and BMI decreased significantly in all participants. Patients were advised to maintain nutritional ketosis, and effective adherence to the protocol allowed the downward trend in body weight and BMI to be maintained throughout the study period (Table 2). A meta-analysis conducted by Bueno et al. [6] compared randomized controlled trials of VLCKD with low-fat diets over one year. The analysis demonstrated a statistically significant advantage in weight reduction for participants following the VLCKD protocol (Table 1). In another study [5], KD (<30 g of carbohydrates per day) was compared with two control groups: one following the standard American diet (SAD) without exercise, and another following SAD with 3–5 sessions of 30 min exercise per week over 10 weeks. The study included 30 adult participants previously diagnosed with metabolic syndrome. After 10 weeks, the ketogenic diet group showed statistically significant improvements (*p* = 0.001) in body weight, body fat percentage, BMI, HbA1c, and ketone levels. All analyzed parameters improved to a greater extent in the KD group compared to both exercise and non-exercise control groups, with five out of seven assessed variables reaching statistical significance. These findings indicate that a controlled diet ketosis approach may represent a highly effective intervention to manage metabolic disturbances associated with metabolic syndrome. Obesity management relies on multiple strategies, initially focusing on lifestyle modification, behavioral interventions, and psychotherapy. Pharmacotherapy may be introduced to support weight control when necessary. If these approaches are insufficient, patients may be considered for bariatric (surgical) treatment [10]. The etiology of obesity is multifactorial and is not yet fully understood. Contributing factors include genetic, environmental, psychological, dietary, and metabolic influences, all of which affect the regulation of body weight, as well as the function and distribution of adipose tissue [31]. Social and environmental factors, such as easy access to inexpensive, palatable, and heavily marketed ultra-processed foods, can promote weight gain. Reduced physical activity combined with a chronic positive energy balance favors adipocyte proliferation and increases overall body mass [1]. However, some studies [2] question the causal role of sedentary behavior in the rising prevalence of obesity. Although the evidence supporting physical inactivity as the primary cause of obesity is limited, a lack of exercise contributes to metabolic disturbances associated with obesity [1]. Despite shared environmental conditions, some individuals develop severe obesity, while others maintain a healthy body weight. Evidence from family, twin, and adoption studies suggests that genetic factors may account for 40–70% of body weight variability [37]. Both genetic and epigenetic variability contribute to obesity development by influencing metabolic pathways, neuronal regulation, and appetite centers. Mutations may be inherited in an autosomal dominant or recessive manner. Epigenetic changes are more complex; they can occur at any point in life and may also be inherited, contributing to obesity risk [3]. Among the most commonly used ketogenic diet protocols for the treatment of obesity, two main types are distinguished based on total caloric intake. The Low-Calorie Ketogenic Diet (LCKD) typically provides 800–1200 kcal/day, whereas VLCKD or VLEKT provides less than 800 kcal/day. VLCKD is characterized by protein intake of 1.2–1.4 g/kg of ideal body weight (approximately 75 g/day), very low carbohydrate content (30–50 g/day, 5–10% of total energy), and a fixed fat intake of around 20 g/day (15–30% of energy), primarily from olive oil [10,22]. Total caloric intake is not rigidly fixed and should be individually tailored to patient needs. Differences between standard ketogenic diets and very low-calorie ketogenic diets are summarized in Table 3. Studies [3,37] demonstrate that short-term ketogenic diet interventions provide a rapid and effective approach for body weight reduction and visceral fat loss. These interventions improve anthropometric parameters, including BMI, waist and hip circumference, and body fat percentage, while maintaining cardiorespiratory fitness. KD also enhances skeletal muscle insulin sensitivity in individuals with obesity, which may reduce the risk of developing type 2 diabetes, a condition closely associated with obesity [8]. A randomized crossover study [8] involving 11 participants with obesity compared two 3-week dietary interventions: a ketogenic diet and a standard diet (SD). Skeletal muscle insulin sensitivity was assessed by the rate of glucose disposal (Rd) during a hyperinsulinemic–euglycemic clamp (HEC), with ΔRd representing the change in glucose disposal rate. ΔRd was higher following the ketogenic diet compared to the standard diet. These results, along with a 2.2 kg reduction in body weight, help explain some of KD’s effects on glucose and lipid metabolism: the ketogenic diet increased glucose uptake during hyperinsulinemia, reduced basal endogenous glucose production and fasting glucose levels, and attenuated the suppression of lipolysis under hyperinsulinemic conditions. Recent studies [38] indicate that VLEKT may be applied in the management of eating disorders, such as binge eating and food addiction, inducing symptom remission. VLEKT combines obesity treatment with stress reduction by exerting short-term positive effects on the sympathetic nervous system and the hypothalamic–pituitary–adrenal (HPA) axis, leading to a significant decrease in salivary cortisol levels [39]. The rapid weight changes observed with VLEKT also facilitate early reductions in systemic inflammation [40]. Supplementation with ω-3 fatty acids is particularly important for patients with obesity, as it supports reductions in visceral fat and total fat mass while preserving lean body mass [41].

### 3.6. Mechanisms of Weight Reduction Associated with the Ketogenic Diet

The ketogenic diet can effectively support weight loss in the short and medium term, particularly in real-life conditions. Although the mechanisms underlying this effect have not yet been fully elucidated, evidence from the literature [7,11,42] indicates several likely physiological processes. The spontaneous reduction in energy intake observed may result from the high satiating potential of protein, modulation of appetite-regulating hormones, and the potential anorexigenic effect of ketone bodies, or from the combined influence of these factors. Some data suggest a small but statistically significant increase in resting energy expenditure. The ketogenic diet promotes improved lipolysis, reflected by a greater dependence on fat as an energy source [7,11,42]. Proteins provide greater satiety than carbohydrates or fats. In ketogenic diets, increased protein intake may play an important role in reducing overall caloric consumption [11,43]. Studies conducted by Lodi et al. [9] indicate that ketone bodies can directly suppress appetite, although the mechanism of this effect has not been fully clarified. Standard weight-loss diets are based on caloric restriction. It is typically recommended that daily energy intake be reduced by 500 to 1000 kcal below body requirements. Although caloric deficit leads to weight loss, it simultaneously increases feelings of hunger and, consequently, appetite. Strategies are recommended to manage hunger and appetite (such as drinking more water, increasing fiber intake, eating more slowly, and choosing solid foods over liquid ones). However, this approach overlooks the fact that long-term caloric restriction triggers physiological mechanisms aimed at restoring energy balance. The average person attempting to lose weight by maintaining a negative energy balance experiences hunger and fatigue, which often leads to cravings for unhealthy foods [44]. Research [45,46] showed that despite weight loss, the ketogenic diet significantly suppresses hunger and reduces ghrelin secretion. The mechanisms underlying this phenomenon have not yet been fully understood, but the ketone bodies themselves may play an important role. The high fat content of a low-carbohydrate ketogenic diet (LCKD) slows gastric emptying, contributing to prolonged satiety [40]. Research by Roekenes and Martins [47] shows that higher levels of β-hydroxybutyrate have been associated with a smaller increase in ghrelin, reduced hunger, and improved levels of satiety hormones. People on a ketogenic diet may be able to lose weight without counting calories, as high levels of satiety reduce snacking and overeating [44]. A report [7] comparing the effects of changes in blood glucose and ketosis on appetite, executive function, and mood in women following two versions of the ketogenic diet (ad libitum and a restrictive Mediterranean ketogenic diet) demonstrated a significant negative correlation between BHB concentration and appetite and subjective desire to eat. A positive correlation was observed between BHB concentration and perceived satiety. The ketogenic diet had stronger effects on reducing appetite than the Mediterranean diet. Leptin is the hormone of satiety produced mainly by white adipose tissue. Obese individuals often show resistance to leptin, resulting in the body producing more leptin despite its reduced physiological effect. The brain’s response to leptin is inhibited by the chronic inflammation characteristic of obesity. The ketogenic diet effectively reduces this inflammation. The reduction in leptin resistance may be one of the mechanisms through which the ketogenic diet exerts its effects. When the body becomes more sensitive to leptin, smaller amounts of this hormone elicit a stronger satiety response, allowing obese individuals to eat their fill while still losing weight [44]. The preservation of weightless body mass has been an important aspect of weight loss. Ketogenic-based diets have been shown to help preserve muscle mass despite increased gluconeogenesis. With low-calorie diets, a loss of approximately 1 kg may occur, with the bone mineral content remaining unchanged. Supplementation with magnesium, calcium, and vitamin D is recommended, as well as adequate protein intake (1–1.5 g/kg/day) and hydration (at least 2 L/day) [22]. In the study by Ebbeling et al. [48], 21 overweight or obese participants, after an initial weight reduction of 10–15% achieved through a preliminary diet, were randomly assigned to one of three diets: a low-fat (LF) diet providing 60% of energy from carbohydrates, 20% from fats, and 20% from proteins (high glycemic load); a low-glycemic index diet providing 40% of energy from carbohydrates, 40% from fats, and 20% from proteins (moderate glycemic load); or a very low-carbohydrate (VLC) diet, providing 10% of energy from carbohydrates, 60% from fats, and 30% from proteins (low glycemic load). After weight loss, when consuming the same calorie content, total daily energy expenditure was approximately 300 kcal/day higher on the VLC diet compared to the LF diet, suggesting that the ketogenic diet may affect the body’s energy expenditure.

### 3.7. The Impact of the Ketogenic Diet on Adipose Tissue and Fat Metabolism in Obesity

Adipose tissue is a vital organ that functions as an energy reservoir, regulates metabolic processes, and secretes hormones; disruptions in its function contribute to the development of metabolic diseases [10]. Evidence from studies [49,50] indicates that the ketogenic diet is effective in the management of lipedema, as it reduces pathological fat accumulation and alleviates pain. Furthermore, VLEKT appears to be a particularly effective intervention, outperforming other dietary strategies—such as the Mediterranean diet and intermittent fasting—in cases of lipedema coexisting with obesity. This diet approach also reduces tumor-related markers, such as survivin, which correlates with improvements in inflammatory status and metabolic health. Survivin is overexpressed in visceral adipose tissue in individuals with obesity, but its expression decreases with weight reduction [51]. VLEKT also increases adiponectin levels, an adipose-derived protein with anti-inflammatory properties [52]. A study [8] evaluated insulin sensitivity in adipose tissue by measuring lipolysis suppression during a hyperinsulinemic–euglycemic clamp (HEC) using a free fatty acid (FFA) tracer. The ketogenic diet was associated with reduced insulin-mediated suppression of lipolysis compared to a standard diet. This finding initially raised concerns about a possible adverse effect of KD on insulin sensitivity, as elevated circulating FFAs can induce insulin resistance through lipotoxic mechanisms. However, such concerns are mitigated within the context of KD: low circulating insulin levels promote the oxidation of excess FFAs and their utilization as an energy substrate, thereby preventing their accumulation in non-adipose tissues. Elevated FFAs can be harmful under hyperinsulinemic conditions, although FFAs alone do not exert this effect in isolation. Research conducted in male Wistar rats [53] demonstrated a protective effect of VLEKT on lipid metabolism, including preservation of insulin sensitivity, enhancement of lipolytic activity, increased thermogenesis in brown adipose tissue, and modulation of the renin–angiotensin system. VLEKT offers short-term benefits in reducing visceral adipose tissue, associated inflammation, and oxidative stress. This diet intervention effectively improves adiposity profiles and may help prevent obesity-related diseases [10].

### 3.8. The Impact of the Ketogenic Diet on Liver Function in Obesity

Ketogenic diets can exert beneficial effects on hepatic function by increasing fatty acid oxidation and reducing circulating insulin levels [10]. Current evidence indicates that KD represents a promising therapeutic strategy for metabolic liver disorders. Its beneficial effects are mediated in part by activating the FGF21–KLB signaling axis in the liver, which plays a crucial role in suppressing de novo lipogenesis. Moreover, KD has been shown to reduce insulin resistance and oxidative stress while improving hepatic antioxidant capacity [54]. VLEKT appears to be particularly effective in supporting hepatic metabolic homeostasis by maintaining redox balance and regulating glucose and lipid metabolism. Findings from recent studies [55] indicate that reductions in hepatic lipofuscin—an established marker of oxidative damage—serve as a key indicator of metabolic improvements during VLEKT. In metabolic liver disorders such as MASLD (Metabolic Dysfunction–Associated Steatotic Liver Disease), VLEKT has demonstrated rapid mobilization and reduction in hepatic fat content, exceeding the results typically observed with standard low-calorie diets [56,57]. Supplementation with vitamin E, omega-3 fatty acids, and silymarin is often recommended as an adjunctive therapy [58]. Reductions in leukocyte and platelet counts observed during KD interventions can indicate attenuation of chronic inflammation associated with hepatic dysfunction, paralleling improvements in liver enzymes such as γ-glutamyltransferase (γGT) [59]. Furthermore, VLEKT appears to influence hepatic inflammation and fibrosis and modulates intercellular communication through extracellular vesicles (EV). Alterations in the profile of small electric vehicles before and after dietary intervention suggest possible regulatory effects on hepatic metabolism at both transcriptomic and lipidomic levels [58,60].

## 4. Discussion

The findings presented in this narrative review provide evidence supporting the effectiveness of the ketogenic diet in the treatment of obesity and metabolic disorders, including type 2 diabetes mellitus. Studies have shown that a 12-week low-calorie, very -low-carbohydrate ketogenic diet improves metabolic parameters such as glucose, insulin, HbA1c, triglycerides, HDL-C, and HOMA-IR, while standard diets often do not lead to significant changes [61]. Ketogenic diets combined with resistance training can enhance fat mass reduction while preserving lean body mass (LBM), in contrast to resistance training alone, which primarily increases muscle mass without significant fat loss [62]. Cannataro et al. [63] demonstrated that circulating microRNAs may serve as biomarkers for low-carbohydrate dietary interventions and reflect regulatory mechanisms of metabolic networks, suggesting a possible epigenetic mechanism underlying the effects of KD [63]. Pilot studies and systematic reviews indicate that low-carbohydrate, high-fat (LCHF) or Mediterranean-style ketogenic diets can reduce body weight, alleviate pain, improve quality of life, and exert anti-inflammatory effects in women with lipedema [64,65,66]. Despite their documented efficacy, ketogenic diets can cause short- and long-term adverse effects. Initial adaptation may trigger the “keto flu,” including fatigue, headache, dizziness, nausea, and gastrointestinal disturbances. Long-term adherence without appropriate supplementation can result in deficiencies in vitamins and minerals (e.g., thiamine, folate, vitamins A and E, calcium, magnesium, iron, potassium) and unfavorable changes in lipid profiles. The risk of nephrolithiasis and hepatic dysfunction has also been reported [14,67,68,69,70,71,72,73,74]. However, weight loss during KD may also be accompanied by reductions in fat-free mass and LBM. Systematic reviews and controlled trials have documented muscle mass loss despite comparable energy intake and activity levels, highlighting the need to tailor interventions to preserve LBM [75,76,77]. VLEKT may be effective for weight reduction; however, simultaneous loss of fat-free mass, which may reflect a reduction in muscle mass, is common and can potentially trigger a yo-yo effect upon returning to a normal diet. Therefore, muscle mass loss represents a significant concern, and VLEKT should not be considered a first-line therapeutic option for obesity management without strategies to preserve muscle mass and proper monitoring [76,78].

In summary, ketogenic diet-based interventions, applied as standalone approaches or in combination with physical activity, have demonstrated effectiveness in promoting weight loss, improving glycemic control, and optimizing body composition. Personalization of ketogenic diet strategies is essential to maximize therapeutic efficacy, minimize potential risks, and improve long-term adherence, particularly among individuals with obesity, type 2 diabetes mellitus, or adipose tissue-related disorders.

## 5. Limitations and Future Directions

The current evidence on ketogenic diets for obesity and T2DM is limited by heterogeneity in study design, population characteristics, and methodological quality. Many studies do not define obesity or T2DM-related endpoints as primary outcomes, which limits generalizability [23,31]. Long-term safety, tolerability, and adherence, particularly for very-low-energy ketogenic therapy, remain insufficiently studied and require careful monitoring of nutrient intake, renal and thyroid function, and organ-specific results [31,43]. The long-term impact of KD on gut health, hepatic function, cardiovascular risk, and preservation of lean body mass remains unclear. VLEKT may lead to rapid weight reduction but is often accompanied by loss of fat-free and lean mass, which can trigger a yo-yo effect, highlighting the importance of muscle-preservation strategies and clinical supervision [76,77,78].

Future research should aim to develop standardized KD protocols that maximize fat loss while maintaining lean body mass. Long-term studies are needed to assess safety, tolerability, and adherence across various populations, and to clarify effects on liver, kidney, and cardiovascular function. Investigating epigenetic mechanisms, including microRNA regulation, can help predict individual metabolic responses and distinguish direct KD effects from weight-loss-mediated benefits.

Well-designed long-term randomized controlled trials are essential to establish evidence-based guidelines, optimize muscle-preservation strategies, and expand the applicability of KD in different metabolic conditions and populations of patients.

## 6. Conclusions

The ketogenic diet shows promise in preventing type 2 diabetes by improving blood sugar control and promoting weight loss, although current clinical evidence is not strong enough to confirm its preventive effects. Short-term use of VLCKD effectively reduces body weight and visceral fat, mainly by decreasing appetite. In people with obesity, it can also reduce inflammation, improve cardiovascular markers, and help regulate the menstrual cycle. However, long-term use can lead to nutritional deficiencies, particularly calcium, which can adversely affect bone health and therefore require individualized dietary planning and appropriate supplementation. VLCKD may positively affect the gut microbiota, but its long-term impact on the digestive system is not yet clear. The ketogenic diet is not recommended during gestational diabetes due to potential nutrient deficiencies and insufficient glucose supply for fetal development. Following the diet can be challenging and usually requires the support of healthcare professionals. Nutrition plans should always be individualized according to clinical guidelines, health conditions, and personal preferences—there is no one-size-fits-all approach. KD is a medical diet and should only be used under the supervision of a medical professional and dietitian, not by healthy individuals without medical need.

## Figures and Tables

**Table 1:** Systematic reviews and meta-analyses on ketogenic diets in adults with type 2 diabetes mellitus.

Type of Analysis	Number of Studies/Participants	KD Definition	Comparator Diets	Main Outcomes	Conclusions	References
Meta-analysis of RCTs	13 RCTs (1577 participants—790 in very low-carbohydrate ketogenic diet, 787 in control groups)	VLCKD	low-fat diets	↓ body weight, ↓ triglycerides, ↑ HDL-C, ↑ LDL-C, ↓ DBP	VLCKD is more effective than LFD for the short-term reduction in weight, TAG, and DBP, but increases LDL-C and HDL-C, requiring further assessment of cardiovascular safety.	[6]
Meta-analysis of RCTs	8 RCTs (611 participants—331 in the ketogenic diet group, others in control groups)	KD/VLCKD (<50 g of carbohydrates/day)	non-ketogenic control diets (>50 g of carbohydrates/day)	↓ body weight, ↓ waist, ↓ HbA1c, ↓ TG, ↑ HDL	KD improved weight, waist, HbA1c, TG, HDL; no major changes in BMI, glucose, insulin, HOMA-IR, cholesterol; mainly short-term.	[28]
A Narrative Review of RCTs	5 RCTs/248 participants	VLCKD: 30–50 g of carbohydrates/day, 60 g of protein, 130 g of fat, ~1500 kcal/day WFKD: 20–50 g carbohydrates/day, protein 1.5 g/kg, fat-free rest	standard low-calorie diet, moderate-carbohydrate calorie-restricted diet, low-fat ADA plate method diet, Mediterranean diet	VLCKD/WFKD → more weight loss, better glycemic control (HbA1c), ↓ triglycerides, possible ↑ LDL	Ketogenic diets improve glycemia and weight in T2DM, might reduce medication, but are difficult to maintain long-term and require supervision.	[31]
Meta-analysis/systematic review	13 studies/5 participants *	KD with very low carbohydrate intake (<55 g/day), high fat, and moderate protein aimed at inducing ketosis; specific macronutrient ratios varied between studies	standard/low-fat diabetes diet or pre-/post-intervention comparison (patients served as their own controls)	improve glycemic control, ↓ body weight and use of medications, ↓ triglycerides, may ↑ LDL	The ketogenic diet reduces fasting glucose and HbA1c, improves lipid metabolism, reduces BMI and body weight, and supports the management of type 2 diabetes.	[32]
Systematic review/meta-analysis of RCTs	11 RCTs/19 reports; total participants not specified	KD (low- carbohydrate, high-fat diet; exact macros not specified)	control diets (low-fat or standard diabetic diets)	no significant differences in glycemic control or weight loss, ↑ HDL, ↓ triglycerides	KD may improve the lipid profile, but shows no additional benefit for glycemic control or weight loss compared to control diets over up to 2 years.	[19]

* Some studies were RCTs that compared KD with control diets (low-fat or standard diabetic diet). Others used a pre-/post-intervention design, where patients served as their own controls for glucose, HbA1c, lipids, and body measurements. Abbreviations: ADA—American Diabetes Association; BMI—Body Mass Index; DBP—Diastolic Blood Pressure; HbA1c—Glycated Hemoglobin; HDL-C—High-Density Lipoprotein Cholesterol; KD—Ketogenic Diet; LDL-C—Low-Density Lipoprotein Cholesterol; LFD—Low-Fat Diet; TG—Triglycerides; VLCKD—Very Low-Carb Ketogenic Diet; WFKD—Well-Formulated Ketogenic Diet.

**Table 2:** Randomized controlled trials evaluating ketogenic diets in adults with type 2 diabetes mellitus.

Study Design	Participants (KD/Control)	Duration	KD Protocol	Control Diet	Conclusions/Main Clinical Outcomes	References
Controlled clinical trial	64 obese adults (BMI > 30) with T2DM 31 participants with high blood glucose levels (diabetes/impaired glucose tolerance)/33 participants with normal blood glucose levels (metabolic control group).	56 weeks	ketogenic diet (<20 carbohydrates/day)	conventional diet	↓ body weight in both groups, ↓ total cholesterol and LDL cholesterol levels, ↑ HDL cholesterol levels and the HDL/LDL ratio, ↓ triglyceride levels, ↑ blood glucose levels in both groups, ↓ in urea levels, long-term KD improves glycemic control in obese patients with type II diabetes and may reduce the need for medication.	[4]
RCT	84 (LCKD/LGID) 49 completed (58.3%)	24 weeks	LCKD (<20 g carbohydrates/day), group meetings, dietary supplements, exercise guidance	reduced-calorie low-glycemic diet (500 kcal/day deficit), group meetings, dietary supplements, exercise guidance	LCKD led to greater improvements in HbA1c, weight loss, ↑ HDL, and reduction/elimination of antidiabetic meds compared to LGID Lifestyle modification with low-carb diet effectively improves type 2 diabetes.	[34]
RCT	89 (VLCKD/standard low-calorie) 45 VLCKD/44 control	4 months	VLCKD (<50 g carbs/day) combined with lifestyle and behavior modification support	standard low-calorie diet	VLCKD led to greater weight and waist loss, improved HbA1c and glycemic control, with mild side effects decreasing over time.	[35]
Pilot feasibility RCT, online	12/13	32 weeks	VLCKD + lifestyle factors (physical activity, sleep, positive emotions, mindfulness)	ADA “Create Your Plate” online program (low-fat diet)	KD group showed greater HbA1c, weight, and triglyceride reductions, achieving more targets, lower dropout; online delivery may enable broader access to effective self-management of type 2 diabetes.	[36]

Abbreviations: ADA “Create Your Plate”—standard online low-fat diet program; HbA1c—Hemoglobin A1c; LCKD/LGID—Low-Carb Ketogenic Diet/Low-Glycemic Index Diet; VLCKD—Very Low-Calorie Ketogenic diet (<50 g carbohydrate/day).

**Table 3:** Differences between a standard KD and VLEKT, according to [10].

	KD	VLEKT
Fats	70–80% of total calories	40–60% of total calories
Proteins	10–20% of total calories	25–35% of total calories
Carbohydrates	5–10% of total calories	5–10% of total calories
Caloric dietary intake	calculated based on total energy consumption	very low calorie (700–800 kcal/day)

## Data Availability

No new data were created or analyzed in this study. Data sharing is not applicable to this article.

## Abbreviations

The following abbreviations are used in this manuscript:

AID	Automated Insulin Delivery
AcAc	Acetoacetate
BHB	Beta-hydroxybutyrate
FFA	Free Fatty Acids
GH	Growth Hormone
IGF-1	Insulin-like Growth Factor
KD	Ketogenic Diet
LCT	Long Chain Triglycerides
LH	Luteinizing Hormone
MAD	Modified Atkins Diet
MCT	Medium Chain Triglycerides
NGSP	National Glycohemoglobin Standardization Program
VLCKD	Very-Low-Calorie Ketogenic Diet
